# Characterization of the abiotic drivers of abundance of nearshore Arctic fishes

**DOI:** 10.1002/ece3.7940

**Published:** 2021-07-22

**Authors:** Noah S. Khalsa, Kyle P. Gatt, Trent M. Sutton, Amanda L. Kelley

**Affiliations:** ^1^ School of Marine and Atmospheric Sciences Stony Brook University Stony Brook NY USA; ^2^ College of Fisheries and Ocean Sciences University of Alaska Fairbanks Fairbanks Alaska USA

**Keywords:** Arctic, climate change, fine resolution, fish abundance, fish communities, physicochemical environment

## Abstract

Fish are critical ecologically and socioeconomically for subsistence economies in the Arctic, an ecosystem undergoing unprecedented environmental change. Our understanding of the responses of nearshore Arctic fishes to environmental change is inadequate because of limited research on the physicochemical drivers of abundance occurring at a fine scale. Here, high‐frequency in situ measurements of pH, temperature, salinity, and dissolved oxygen were paired with daily fish catches in nearshore Alaskan waters of the Beaufort Sea. Due to the threat that climate change poses to high‐latitude marine ecosystems, our main objective was to characterize the abiotic drivers of abundance and elucidate how nearshore fish communities may change in the future. We used generalized additive models (GAMs) to describe responses to the nearshore environment for 18 fish species. Relationships between abundance and the physicochemical environment were variable between species and reflected life history. Each abiotic covariate was significant in at least one GAM, exhibiting both nonlinear and linear associations with abundance. Temperature was the most important predictor of abundance and was significant in GAMs for 11 species. Notably, pH was a significant predictor of abundance for six species: Arctic cod (*Boreogadus saida*), broad whitefish (*Coregonus nasus*), Dolly Varden (*Salvelinus malma*), ninespine stickleback (*Pungitius pungitius*), saffron cod (*Eleginus gracilis*), and whitespotted greenling (*Hexagrammos stelleri*). Broad whitefish and whitespotted greenling abundance was positively associated with pH, while Arctic cod and saffron cod abundance was negatively associated with pH. These results may be a bellwether for future nearshore Arctic fish community change by providing a foundational characterization of the relationships between abundance and the abiotic environment, particularly in regard to pH, and demonstrate the importance of including a wider range of physicochemical habitat covariates in future research.

## Graphical abstract

Our understanding of the responses of nearshore Arctic fishes to environmental change is inadequate because of limited research on physicochemical drivers of abundance occurring on short time scales. Here, high‐frequency in situ measurements of pH, temperature, salinity, and dissolved oxygen were paired with daily fish catches in the nearshore Beaufort Sea, Alaska. We found that abiotic drivers of abundance were inconsistent between species, with potential implications for fish communities under future climate change.

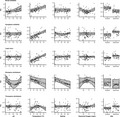

## 1 INTRODUCTION

Unprecedented environmental change is occurring faster in the Arctic than the global average and is characterized by warming waters (Chan et al., 2019), ocean acidification (Qi et al., 2017), sea ice retreat (Bonsell & Dunton, 2018), deoxygenation (Bopp et al., 2013), and ocean freshening (Shu et al., 2018). Such changes threaten the ability for fishes to function optimally and, ultimately, may act to destabilize coastal marine ecosystems (Chan et al., 2019; Overland et al., 2014; Qi et al., 2017; Reist et al., 2006; Wassmann et al., 2011). Notably, nearshore Arctic fishes support socioeconomically vital subsistence fisheries (Zeller et al., 2011) and play roles as keystone species in marine ecosystems (Reist et al., 2006). As changes to the physicochemical environment continue to occur, nearshore Arctic fishes will need to adapt, evolve, or migrate, to mitigate deleterious impacts to physiology and behavior, potentially leading to shifts in distribution and abundance, which may result in fish community restructuring (Beever et al., 2017; Reist et al., 2006). Indeed, mounting evidence shows that ongoing nearshore Arctic fish community change is correlated with a warming environment (Gatt et al., 2019; Priest, 2020; Priest et al., 2018).

Arctic fishes are particularly susceptible to environmental change because their environmental niche is attenuated due to the direct and indirect effects of lower seawater temperature when compared to fishes from southern latitudes (Reist et al., 2006). Specific temperature ranges (the difference between lower and upper temperature tolerance thresholds) decrease toward high and low latitudes, with the most resilient fishes occupying temperate freshwater ecosystems (Dahlke et al., 2020). Potential impacts of climate change to key life‐history aspects, such as reproduction and growth rate, could significantly hamper the ability for species to persist under climate change (Dahlke et al., 2020). Indeed, it is imperative to understand the response of Arctic fishes to their physicochemical environments in order to assess the potential consequences of climate change.

Describing how the physicochemical environment influences the abundance and habitat use of Arctic fishes is important for establishing a baseline record of nearshore assemblages so that future impacts of climate change can be determined. In their review of climate change impacts on Arctic fishes, Reist et al. (2006) stated: “Other than logical extrapolations, most responses to climate change are impossible to quantify due to the absence of basic physiological information for most Arctic fish species and the incomplete understanding of the overall associations of ecological processes with present‐day climate parameters.” Despite the dire need for research understanding physiological and ecological impacts of climate change on Arctic fishes, there is still a paucity of data regarding these relationships in the nearshore Arctic, particularly with respect to carbonate chemistry.

The majority of the published literature relating the physicochemical environment to the abundance of Arctic fishes in order to understand the impacts of climate change is limited spatially, temporally, and taxonomically. Studies rely almost exclusively on data collected from long‐term trawl surveys conducted in offshore waters (Iken et al., 2019; Logerwell et al., 2018; Mueter & Litzow, 2008). Trawl surveys collect point source data at a coarse temporal resolution (as opposed to high‐frequency oceanographic measurements); thus, they cannot account for changes in the nearshore environment transpiring on fine temporal scales. Even so, these studies are important for understanding long‐term ecological changes occurring offshore. What is more, trawl surveys are, by the nature of the collection method, biased toward epibenthic fishes and often do not capture highly migratory nearshore fishes such as whitefishes of the genus *Coregonus*, which constitute a major portion of coastal fish taxa (Gatt et al., 2019; Griffiths et al., 1998; Priest, 2020; Zeller et al., 2011). Utilizing gears deployable in the nearshore that can be sampled at higher frequencies and capture highly mobile species (e.g., fyke nets) can address these data gaps. Several Arctic fish studies have begun to close these knowledge gaps by sampling in the nearshore; nonetheless, they are primarily focused on species deemed most important for subsistence fisheries such as Arctic cisco (*Coregonus autumnalis*), broad whitefish (*Coregonus nasus*), Dolly Varden (*Salvelinus malma*), and least cisco (*Coregonus sardinella*) (Gatt et al., 2019; Griffiths et al., 1992, 1998; Priest, 2020; Priest et al., 2018) and are temporally limited by focusing on environmental correlates of interannual changes in fish abundance (Griffiths et al., 1992). Lastly, studies thus far have concentrated almost exclusively on the impacts of temperature and salinity on the distribution and abundance of Arctic fishes, with none, to our knowledge, incorporating pH as a potential habitat covariate.

The measure of pH is seldom included in ecological studies of fishes as it is believed that fishes are resilient to ocean acidification due to active ion transport systems which act to buffer against mild acidosis (Cattano et al., 2018; Munday et al., 2013). It is also logistically difficult, expensive, and requires specialized equipment and skills to accurately measure pH, which are barriers only exacerbated by working in the Arctic (Miller et al., 2018). Despite the common conjecture that ocean acidification will have little effect on fishes relative to other climate change impacts, emerging work suggests that significant long‐term physiological and behavioral impacts will ensue, particularly in regions already experiencing pH levels projected for the year 2100 (Heuer & Grosell, 2014; Kelley & Lunden, 2017). As such, it is important to incorporate pH data into baseline ecological studies and begin characterizing the response of Arctic fishes to ocean acidification for understanding the consequences of climate change, holistically.

Here, we couple high‐frequency in situ time‐series of pH, temperature, salinity, and dissolved oxygen with abundance data for 18 nearshore Arctic fish species with the goals of: (a) characterizing how the abiotic environment influences fish abundance in the nearshore Arctic at a fine temporal scale and (b) evaluating the importance of expanding Arctic fish ecological studies to incorporate a wider range of habitat covariates. We intend for this study to be the first link in a longer chain of understanding about the responses of nearshore fishes to the abiotic environment to elucidate implications of a changing Arctic.

## 2 METHODS

### 2.1 Study site and oceanographic sampling

**FIGURE 1:**
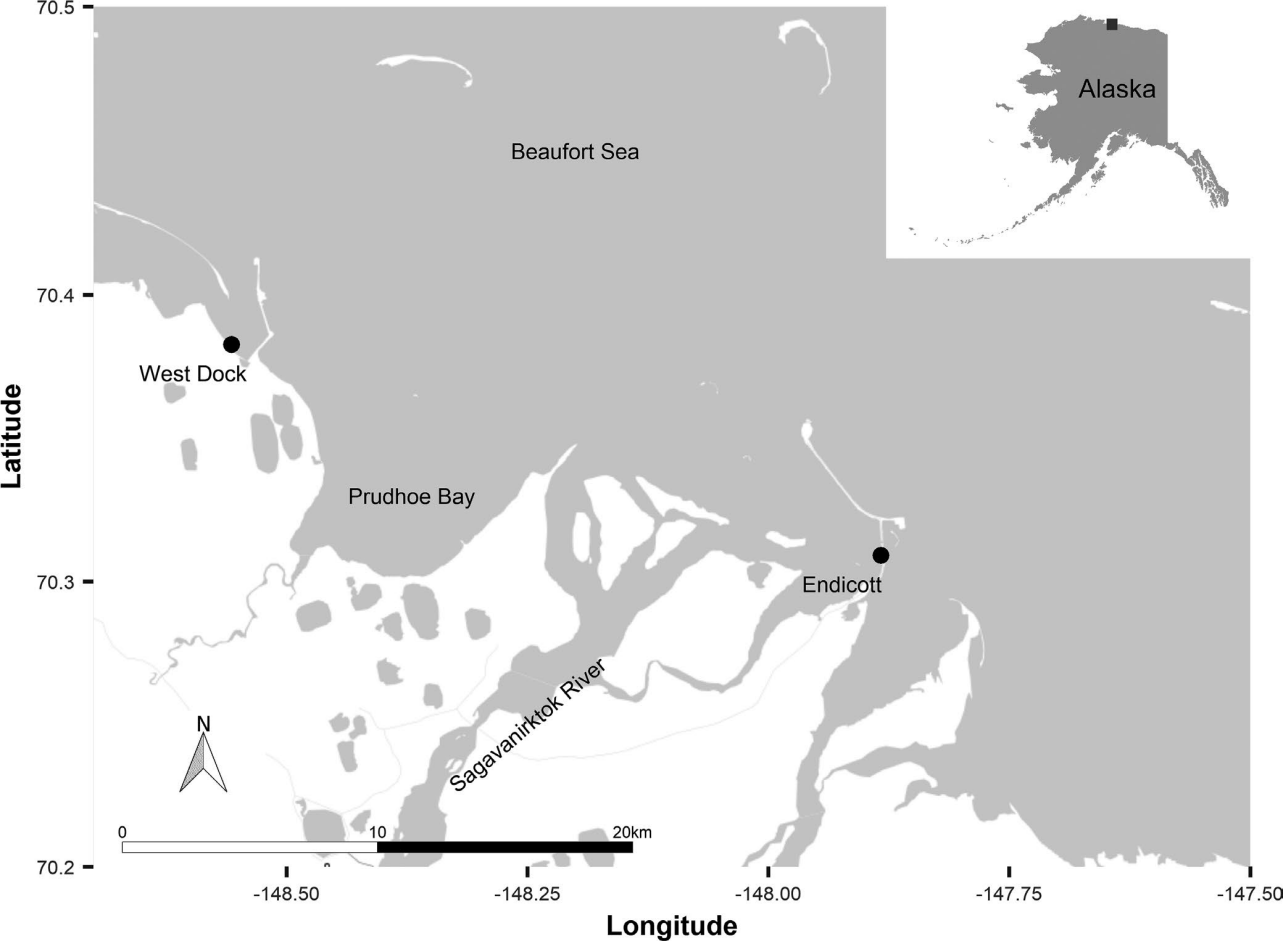
Overview map of our study sites along the coastal Beaufort Sea, Alaska

This study took place at two nearshore sites in the Beaufort Sea, Alaska (Figure 1), during the ice‐free period of the summer of 2019. Research was undertaken in conjunction with the Beaufort Sea Nearshore Long‐Term Fish Monitoring Program (BSNLTFMP) (Gatt et al., 2019; Priest et al., 2018), with our sites located at Endicott and West Dock. This study was temporally constrained by the presence of ice at the beginning and the occurrence of adverse weather at the end which truncated the sampling season. Endicott is situated at the mouth of the Sagavanirktok River and has considerable freshwater influence, while West Dock is a primarily marine site, experiencing less freshwater flux relative to Endicott. Endicott and West Dock are separated by approximately 23‐km straight line distance. Nearshore oceanography in the region is known to be driven by wind, freshwater input, and ice formation, notably, processes identified as major contributors to nearshore carbonate chemistry variability (Craig et al., 1982; Miller et al., 2021; Priest et al., 2018). Sites were stationed roughly 100‐m offshore and at a depth of 1–2 m throughout the summer, only reaching peak depths during periods of sustained westerly winds. Westerly winds drive water levels up and retain brackish water inshore, while easterly winds favor offshore upwelling and bring marine water nearshore (Gatt et al., 2019). These sites were chosen because they span the breadth of physicochemical conditions fishes experience in the region—freshwater to marine—which would ensure that our nets would capture both marine and freshwater fishes.

Oceanographic sensor arrays, which included a Sea‐Bird Scientific SeaFET™ pH sensor, PME miniDOT optical oxygen logger, and an Onset HOBO conductivity logger were deployed at each site between 2 July and 21 August 2019. Sensor arrays were secured approximately 0.5 m from the benthos at a depth of roughly 1 m to fyke net anchor poles used for fish sampling. Oceanographic measurements of pH, temperature, salinity, and dissolved oxygen were sampled hourly (UTC). Temperature, salinity, and dissolved oxygen were measured with an average of one sample per frame (a single averaged measurement) and one frame per burst (time interval over which samples are taken), while pH was measured with an average of one sample per frame and 10 frames per burst. Temperature was recorded using the pH sensor's onboard thermistor. We calibrated the SeaFET™ sensors, propagated their error (see Miller & Kelley, 2021), and calibrated the pH, salinity, and dissolved oxygen data according to the best practices in chemical oceanography (for details, see the supplementary file: Environmental Data Processing). Measurements of pH are in total hydrogen ion concentration, and salinity is unitless throughout the manuscript.

### 2.2 Fish sampling

Weather and safe sampling conditions permitting, fish were sampled at each site using paired fyke nets with 1.8 × 1.7 m openings, set side by side. Net openings were oriented toward the shore, and a 60‐m blocker net led to the shore. Blocker wings extended 15 m from each side of the cod‐end net openings. This net setup allowed the capture of fish swimming bidirectionally along the shore. Mesh sizes of 2.5 cm were used for the blocker nets and wings, and the mesh size was 1.27 cm for the fyke nets. Each fyke net had three consecutive throats extending from behind the frames. Fish were identified to species and enumerated (including mortalities) daily at both Endicott and West Dock. Fish were not separated by age or size for this study, although length and supplementary biological samples were collected on select species for unrelated projects. Post‐identification fish were released away from the net openings to reduce the chance of recapture. Fish identification was based on Mecklenberg et al. (2002), George et al. (2009), and Thorsteinson and Love (2016). Endicott was fished from 1 July to 22 August 2019, and West Dock was fished from 6 July to 22 August 2019. All fish collection and handling procedures were approved by the Alaska Department of Fish and Game through Aquatic Resource Permit No. CF‐19‐021(2) and by the UAF Animal Care and Use Committee as assurance 1054743.

Daily fish catch at each site for each species was converted to a catch‐per‐unit‐effort (CPUE) index of abundance to account for potential differences in how long the nets were out. Relative fish abundance data were calculated as the total catch at each sampling site by species. Total catch was then standardized by sampling effort to determine the daily CPUE (fish/hr).

### 2.3 Statistical analyses

To explore the influence of the physicochemical environment on daily fish catch, we used generalized additive models (GAMs) with a Tweedie error family and log link (Wood, 2017). Thin plate regression spline smoothers were used to model relationships between CPUE and the physicochemical covariates (pH, temperature, salinity, and dissolved oxygen). Smooth terms were restricted to three degrees of freedom to limit the models to fitting biologically realistic nonlinear relationships. A fixed‐factor variable for site was included to account for potential site‐specific differences in catch that were not captured by the physicochemical variables. Site was not included in the model if fish were only caught at one site over the sampling period. The GAMs were fit using restricted maximum likelihood within the *mgcv* R package (Wood, 2017). Catch‐per‐unit‐effort data were overdispersed and in some cases zero inflated, which is why a Tweedie error family was chosen. Daily CPUE and daily averages for each environmental covariate were temporally aligned prior to modeling, and data from both sites were pooled. Despite the possibility for hierarchical structure in our data as a consequence of pooling multiple sites, site was not modeled as a random effect because there were less than five sites, which could lead to weak estimates of variance (Harrison et al., 2018). Any days when sampling did not occur due to weather conditions were omitted from modeling. Only values after the first sensor calibration on 4 July 2019 were included in statistical analyses. All environmental covariates were retained for modeling because Pearson's correlations were <0.7 within and between sites and the VIF was <3 (Dormann et al., 2013; Harrison et al., 2018). A variable selection procedure was not carried out. Instead, one holistic model was developed for each species and p‐values (*α* = 0.05) were used to assess the relative importance of each covariate. Residual plots were reviewed using the gam.check function within the *mgcv* package to assess whether model assumptions were met. Plots were generated using the *ggplot2* (Wickham, 2016) and *visreg* (Breheny & Burchett, 2017) packages. All statistical analyses were conducted in R version 4.0.2 (R Core Team, 2018).

## 3 RESULTS

### 3.1 Environmental characterization

**FIGURE 2:**
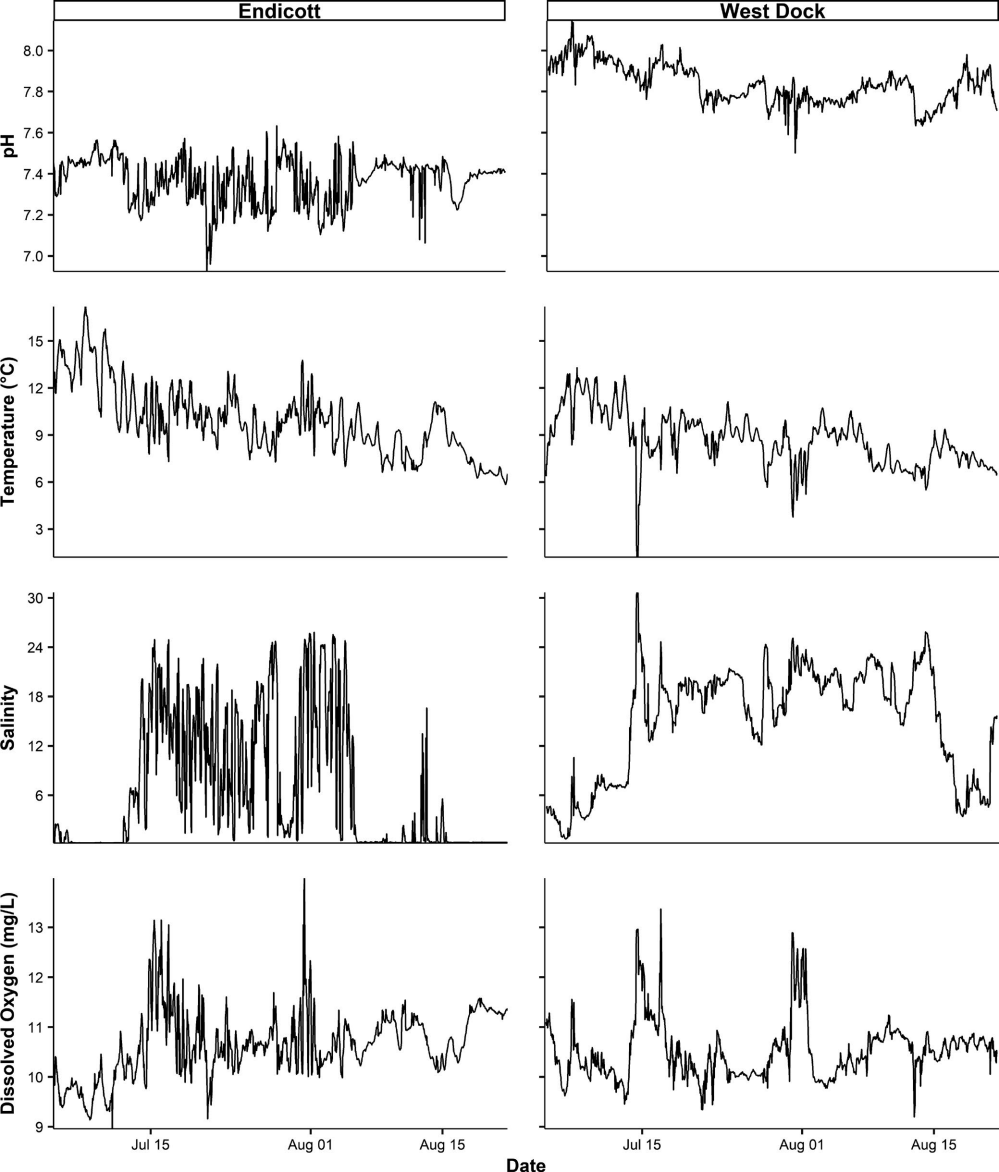
Hourly measurements of pH, temperature, salinity, and dissolved oxygen recorded at Endicott and West Dock between 4 July and 21 August 2019

Endicott was more acidic, fresher, warmer, and more oxygen rich than West Dock (Figure 2; Table 1). Strong significant negative correlations between environmental covariates were observed between pH and salinity (*R* ^2^ = 0.69) and temperature and oxygen (*R* ^2^ = 0.67) at Endicott. At West Dock, strong significant negative correlations were observed between pH and salinity (*R* ^2^ = 0.63) and temperature and oxygen (*R* ^2^ = 0.56). Notable environmental observations for the study included a high temperature of 17.2°C and pH low of 6.9 recorded at Endicott (Table 1).

**TABLE 1:** Summary statistics for hourly measurements of pH, temperature, salinity, and dissolved oxygen recorded at Endicott and West Dock

Measured variable	Endicott			West Dock		
	Mean ± *SD*	Median	Range	Mean ± *SD*	Median	Range
pH	7.4 ± 0.1	7.4	6.9–7.6	7.8 ± 0.1	7.8	7.5–8.1
Temperature (°C)	9.9 ± 2.2	9.8	5.9–17.2	8.7 ± 1.7	8.7	1.2–13.3
Salinity	6.6 ± 8.0	1.7	0.2–25.8	15.1 ± 6.7	17.1	0.7–30.6
Dissolved oxygen (mg/L)	10.7 ± 0.7	10.6	9.0–14.0	10.5 ± 0.6	10.4	9.2–13.4

### 3.2 Catch description

**FIGURE 3:**
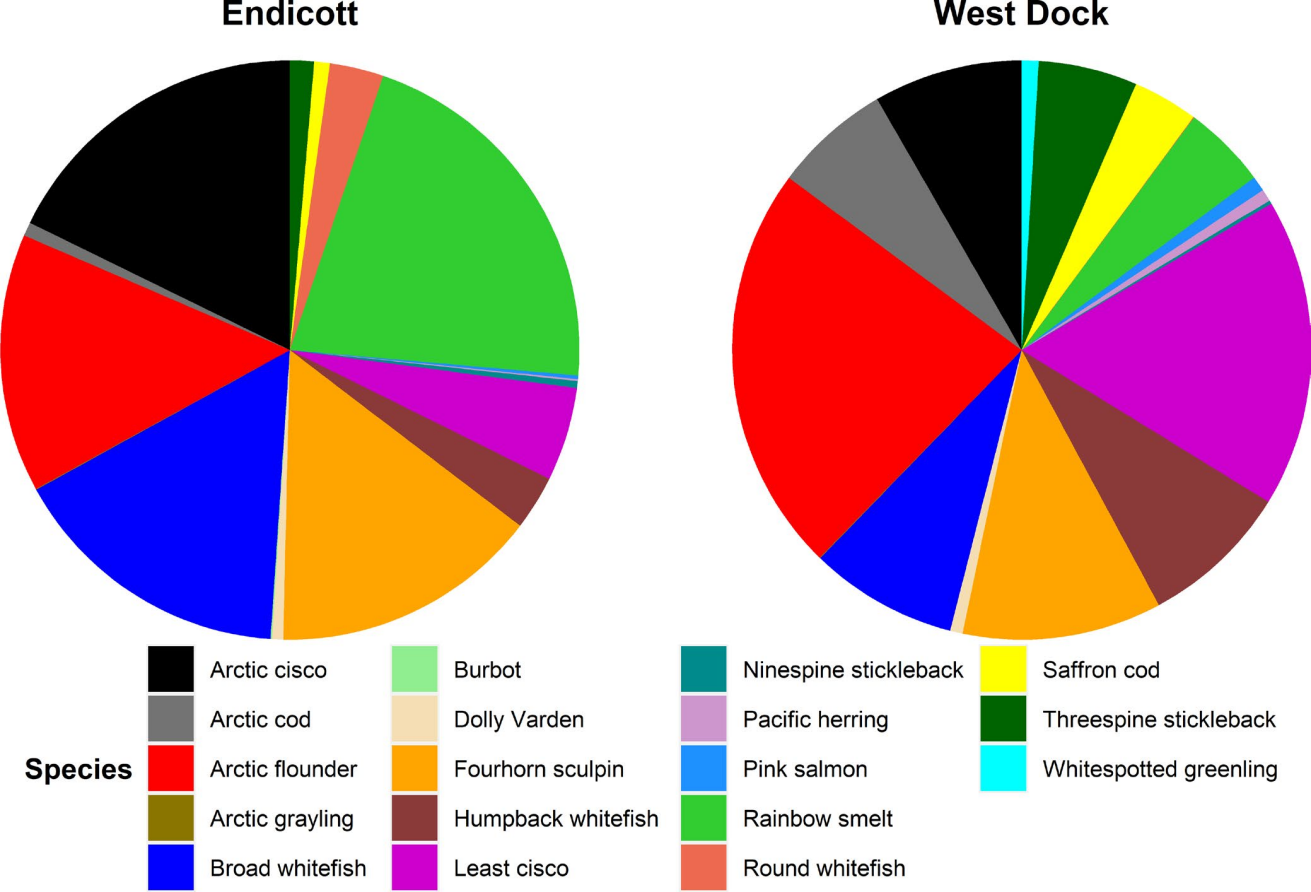
Total CPUE for each species recorded at Endicott and West Dock. Note that these were the total CPUE for the entire sampling period at these sites during 2019, which included sampling prior to and after oceanographic data were being recorded

Eighteen fish species were identified between 4 July and 21 August 2019. Species identified during the study represented diadromous, freshwater, and marine life histories. Five species were amphidromous, four were anadromous, three were freshwater, and six were marine (Table 2). Catches at Endicott were dominated by Arctic cisco and broad whitefish, while catch at West Dock was predominately comprised of Arctic flounder (*Pleuronectes glacialis*) and least cisco (Figure 3). Differences in species composition between sites were evident in the appearance of Arctic grayling (*Thymallus arcticus*), burbot (*Lota lota*), and ninespine stickleback (*Pungitius pungitius*) in much greater numbers at Endicott than West Dock (Figure 3). Similarly, West Dock accounted for a much higher proportion of the catch for Pacific herring (*Clupea pallasii*) and whitespotted greenling (*Hexagrammos stelleri*) (Figure 3).

**TABLE 2:** Species identified during the study period and their associated life history

Life history	Amphidromous	Anadromous	Freshwater	Marine
Species	Broad whitefish	Arctic cisco	Arctic grayling	Arctic cod
	Humpback whitefish	Dolly Varden	Burbot	Arctic flounder
	Least cisco	Pink salmon	Round whitefish	Fourhorn sculpin
	Ninespine stickleback	Rainbow smelt		Pacific herring
	Threespine stickleback			Saffron cod
				Whitespotted greenling

### 3.3 Amphidromous fish GAMs

**FIGURE 4:**
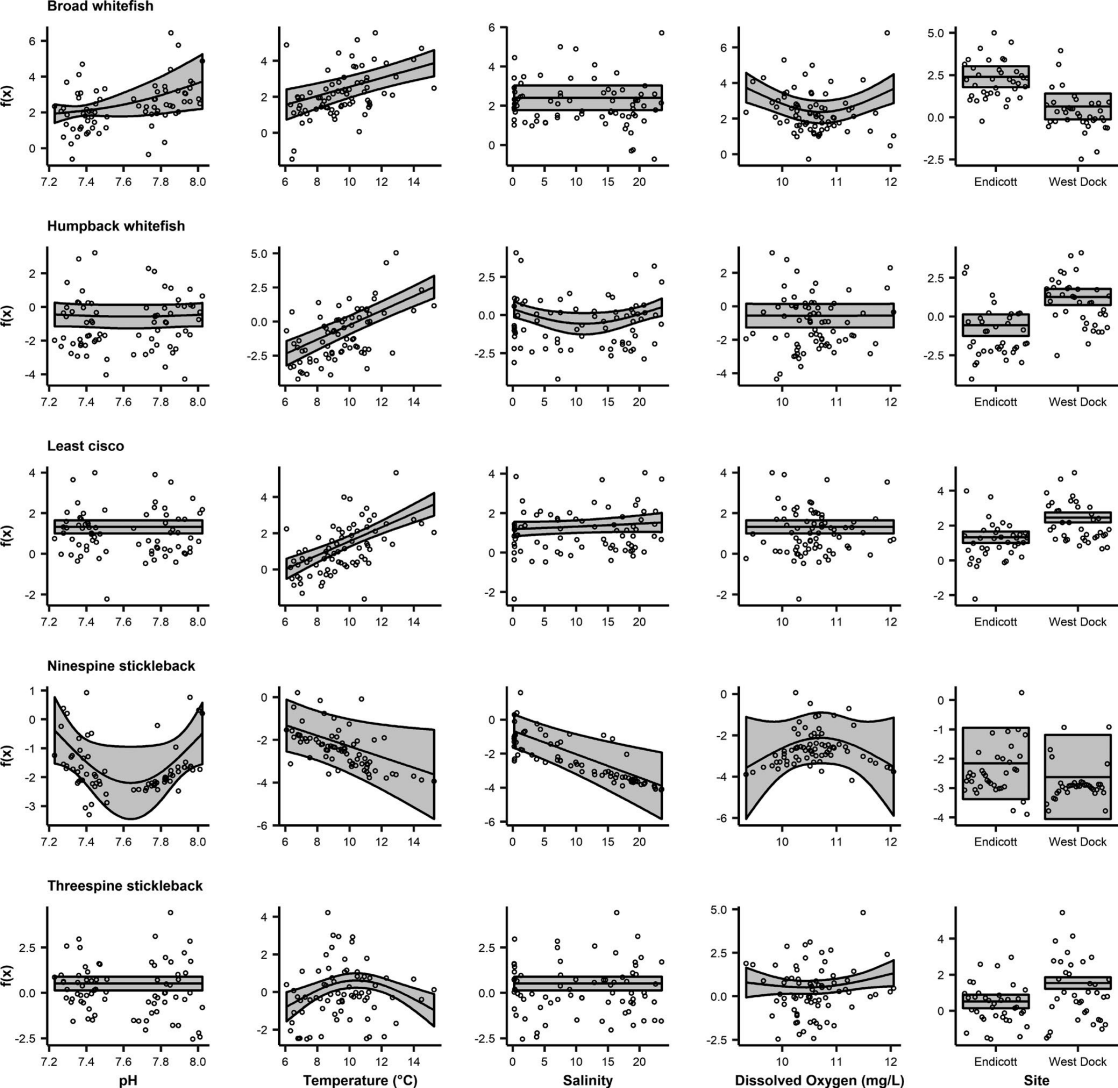
Fits of GAM covariates including 95% confidence bounds for amphidromous species. Note that the environmental covariates were smooth terms while site is parametric

All covariates were significantly associated with CPUE for amphidromous fish (Figure 4; Table 3). The GAM for broad whitefish explained 65% of the variance in CPUE (Table 3). Broad whitefish CPUE was significantly associated nonlinearly with pH, linearly with temperature, and nonlinearly with dissolved oxygen (Figure 4). Catch‐per‐unit‐effort increased with pH and temperature and decreased with dissolved oxygen until reaching an inflection point at ~10.5 mg/L, at which point the relationship became positive (Figure 4). Site significantly influenced catch, as indicated by higher CPUE at Endicott for broad whitefish (Figure 4). Fifty‐six percent of the variance in CPUE for humpback whitefish (*Coregonus pidschian*) was explained by the GAM (Table 3). Humpback whitefish CPUE was significantly associated linearly with temperature and nonlinearly with salinity (Figure 4). Catch‐per‐unit‐effort increased with temperature and decreased with salinity until ~11, when CPUE began increasing with salinity (Figure 4). Humpback whitefish CPUE was significantly higher at West Dock than Endicott (Figure 4). For least cisco, 42% of the variance in CPUE was explained by the GAM (Table 3). Least cisco CPUE increased significantly in a linear manner with temperature (Figure 4). The GAM indicated that least cisco CPUE was significantly greater at West Dock than Endicott (Figure 4). Forty‐three percent of the variance in ninespine stickleback CPUE was explained by the GAM (Table 3). Ninespine stickleback CPUE was significantly associated nonlinearly with pH and linearly with temperature and salinity (Figure 4; Table 3). Catch‐per‐unit‐effort decreased with pH until reaching an inflection point at ~7.6, at which point CPUE increased with pH (Figure 4). Ninespine stickleback CPUE decreased with temperature and salinity (Figure 4). The GAM explained 27% of the variance in CPUE for threespine stickleback (*Gasterosteus aculeatus*) (Table 3). Threespine stickleback CPUE was significantly associated nonlinearly with temperature and dissolved oxygen (Figure 4). Temperature had a positive relationship with CPUE until ~10°C, when CPUE decreased (Figure 4). Threespine stickleback CPUE decreased weakly with dissolved oxygen until ~10.5 mg/L, above which CPUE increased (Figure 4). Catch‐per‐unit‐effort was significantly higher at West Dock for threespine stickleback (Figure 4).

**TABLE 3:** GAM models for all species

Species	Smooth terms				Parametric term	
	pH	Temperature (°C)	Salinity	Dissolved oxygen (mg/L)	Site (ref = West Dock)	Deviance explained
Arctic cisco						
*df*	0.54	0.96	0.00	0.00	—	0.57
Coefficient ± *SE*	—	—	—	—	−0.40 ± 0.36	
*p*‐value	0.23	0.00	0.84	0.00	0.27	
Arctic cod						
*df*	0.89	0.00	0.72	0.95	—	0.61
Coefficient ± *SE*	—	—	—	—	4.84 ± 1.74	
*p*‐value	0.00	0.56	0.06	0.00	0.01	
Arctic flounder						
*df*	0.00	0.97	1.54	0.00	—	0.28
Coefficient ± *SE*	—	—	—	—	0.16 ± 0.23	
*p*‐value	0.99	0.00	0.01	0.38	0.49	
Arctic grayling						
*df*	0.00	0.00	0.69	0.89	—	0.55
Coefficient ± *SE*	—	—	—	—	0.08 ± 1.37	
*p*‐value	0.60	0.49	0.10	0.00	0.95	
Broad whitefish						
*df*	1.39	0.95	0.00	0.93	—	0.65
Coefficient ± *SE*	—	—	—	—	−1.76 ± 0.62	
*p*‐value	0.03	0.00	0.54	0.00	0.01	
Burbot						
*df*	0.58	1.61	0.00	0.70	—	0.66
Coefficient ± *SE*	—	—	—	—	—	
*p*‐value	0.21	0.20	0.95	0.18	—	
Dolly Varden						
*df*	0.90	0.92	0.91	0.00	—	0.34
Coefficient ± *SE*	—	—	—	—	0.17 ± 0.37	
*p*‐value	0.00	0.00	0.00	0.59	0.65	
Fourhorn sculpin						
*df*	0.00	0.98	0.81	1.29	—	0.51
Coefficient ± *SE*	—	—	—	—	−0.31 ± 0.21	
*p*‐value	0.65	0.00	0.02	0.00	0.15	
Humpback whitefish						
*df*	0.22	0.99	0.87	0.01	—	0.56
Coefficient ± *SE*	—	—	—	—	1.82 ± 0.29	
*p*‐value	0.26	0.00	0.01	0.28	0.00	
Least cisco						
*df*	0.00	0.98	0.62	0.00	—	0.42
Coefficient ± *SE*	—	—	—	—	1.14 ± 0.23	
*p*‐value	0.96	0.00	0.13	0.32	0.00	
Ninespine stickleback						
*df*	0.79	0.82	0.93	0.64	—	0.43
Coefficient ± *SE*	—	—	—	—	−0.47 ± 0.53	
*p*‐value	0.03	0.02	0.00	0.15	0.39	
Pacific herring						
*df*	0.00	0.23	0.84	0.68	—	0.23
Coefficient ± *SE*	—	—	—	—	0.65 ± 0.63	
*p*‐value	0.88	0.29	0.02	0.12	0.30	
Pink salmon						
*df*	0.00	0.86	0.85	0.00	—	0.34
Coefficient ± *SE*	—	—	—	—	0.54 ± 0.58	
*p*‐value	0.99	0.01	0.01	0.82	0.36	
Rainbow smelt						
*df*	0.00	1.40	0.98	0.84	—	0.46
Coefficient ± *SE*	—	—	—	—	−2.21 ± 0.34	
*p*‐value	0.76	0.00	0.01	0.01	0.00	
Round whitefish						
*df*	0.60	0.00	0.85	1.25	—	0.70
Coefficient ± *SE*	—	—	—	—	−5.10 ± 0.98	
*p*‐value	0.12	0.46	0.01	0.00	0.00	
Saffron cod						
*df*	1.40	0.69	0.31	0.92	—	0.42
Coefficient ± *SE*	—	—	—	—	2.61 ± 0.89	
*p*‐value	0.00	0.07	0.20	0.00	0.00	
Threespine stickleback						
*df*	0.00	0.93	0.00	1.26	—	0.27
Coefficient ± *SE*	—	—	—	—	1.02 ± 0.23	
*p*‐value	0.96	0.00	0.57	0.04	0.00	
Whitespotted greenling						
*df*	0.94	0.00	0.40	0.84	—	0.79
Coefficient ± *SE*	—	—	—	—	—	
*p*‐value	0.00	0.74	0.24	0.01	—	

Note

Degrees of freedom are for the smooth terms, and coefficients are for the parametric term. Significance levels for each parameter and the model deviance explained are presented.

### 3.4 Anadromous fish GAMs

**FIGURE 5:**
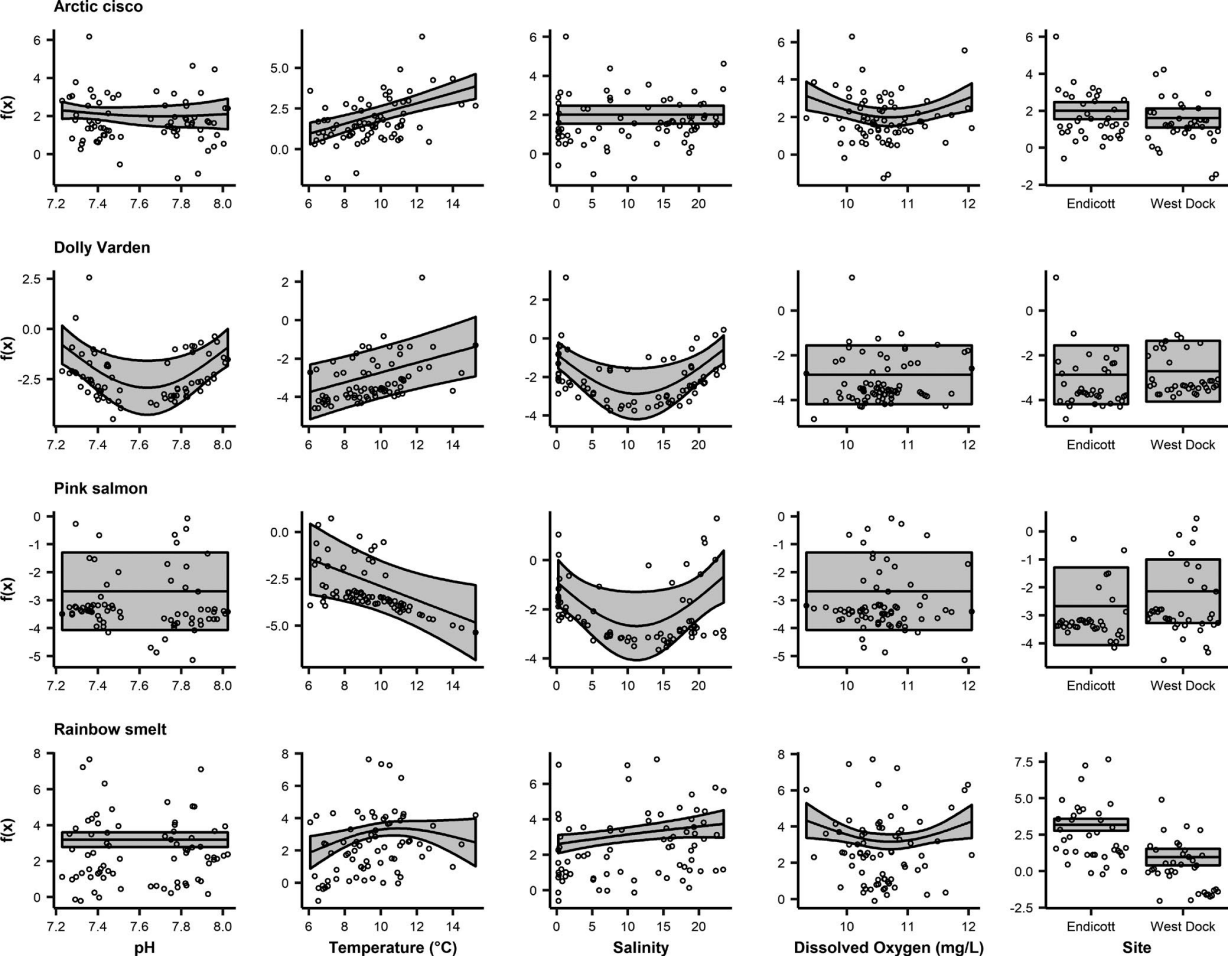
Fits of GAM covariates including 95% confidence bounds for anadromous species. Note that the environmental covariates were smooth terms while site is parametric

Anadromous fish abundance was significantly associated with all covariates (Figure 5; Table 3). Variance in Arctic cisco CPUE (57%) was explained by the GAM (Table 3). Arctic cisco CPUE was significantly associated linearly with temperature and nonlinearly with dissolved oxygen (Figure 5). Catch‐per‐unit‐effort increased with temperature and decreased with dissolved oxygen until reaching ~10.8 mg/L when CPUE began increasing with dissolved oxygen (Figure 5). The GAM for Dolly Varden explained 34% of the variance in CPUE. Dolly Varden CPUE was significantly associated nonlinearly with pH, linearly with temperature, and nonlinearly with salinity (Figure 5). Catch‐per‐unit‐effort decreased with pH until reaching an inflection point at ~7.6 when CPUE began increasing with pH (Figure 5). Temperature and CPUE were positively related (Figure 5). Dolly Varden CPUE decreased with salinity until ~11 when it began increasing with salinity (Figure 5). The variance in pink salmon (*Oncorhynchus gorbuscha*) CPUE (34%) was explained by the GAM (Table 3). Pink salmon CPUE was significantly associated linearly with temperature and nonlinearly with salinity (Figure 5). Catch‐per‐unit‐effort decreased with temperature and salinity until it reached an inflection point at a salinity of ~11 and the relationship became positive (Figure 5). The GAM for rainbow smelt (*Osmerus mordax*) explained 46% of the variance in CPUE (Table 3). Rainbow smelt CPUE was significantly associated nonlinearly with temperature, linearly with salinity, and nonlinearly with dissolved oxygen (Figure 5). Temperature and CPUE were positively related until reaching an inflection point at ~10°C after which the relationship was negative (Figure 5). Rainbow smelt CPUE increased with salinity (Figure 5). Catch‐per‐unit‐effort was negatively related to dissolved oxygen until ~10.8 mg/L when the relationship became positive (Figure 5). Endicott had significantly higher rainbow smelt CPUE than West Dock (Figure 5).

### 3.5 Freshwater fish GAMs

**FIGURE 6:**
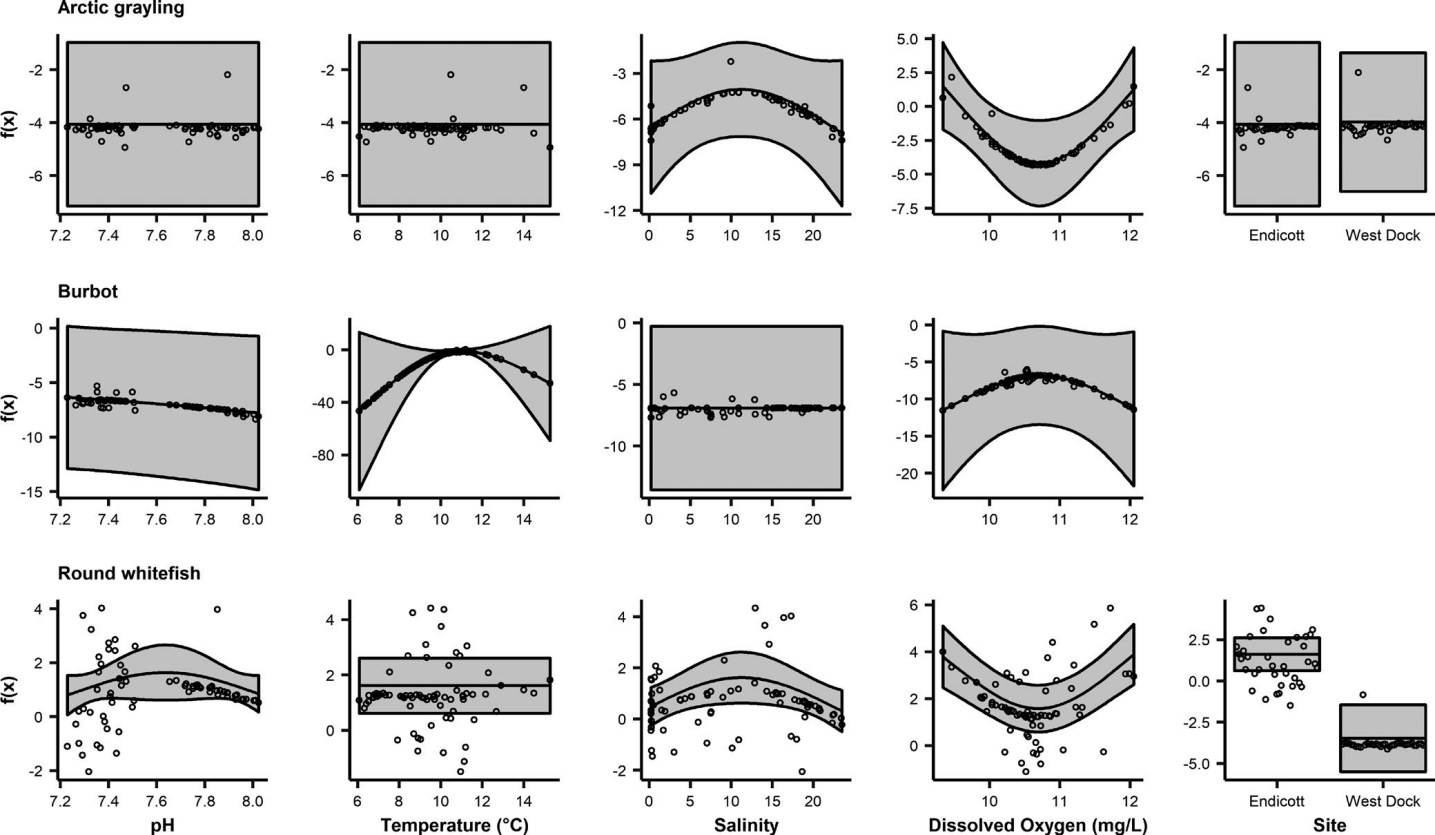
Fits of GAM covariates including 95% confidence bounds for freshwater species. Note that the environmental covariates were smooth terms while site is parametric

Abundance of freshwater fish was associated with salinity, dissolved oxygen, and site (Figure 6; Table 3). Variance in Arctic grayling CPUE (55%) was explained by the GAM. Arctic grayling CPUE was significantly nonlinearly associated with dissolved oxygen, exhibiting a negative association until ~10.8 mg/L, above which the relationship became positive (Figure 6). The GAM for burbot explained 66% of the variance in CPUE, but none of the covariates were significant in the model (Table 3). The variance in round whitefish (*Prosopium cylindraceum*) CPUE (70%) was explained by the GAM (Table 3). Round whitefish CPUE was significantly associated nonlinearly with salinity and dissolved oxygen (Figure 6). Salinity and CPUE were positively related until ~10 when the relationship became negative (Figure 6). Round whitefish CPUE decreased with dissolved oxygen until reaching an inflection point at ~10.8 mg/L, above which it increased with dissolved oxygen (Figure 6). Catch‐per‐unit‐effort was significantly greater at Endicott than West Dock for round whitefish (Figure 6).

### 3.6 Marine fish GAMs

**FIGURE 7:**
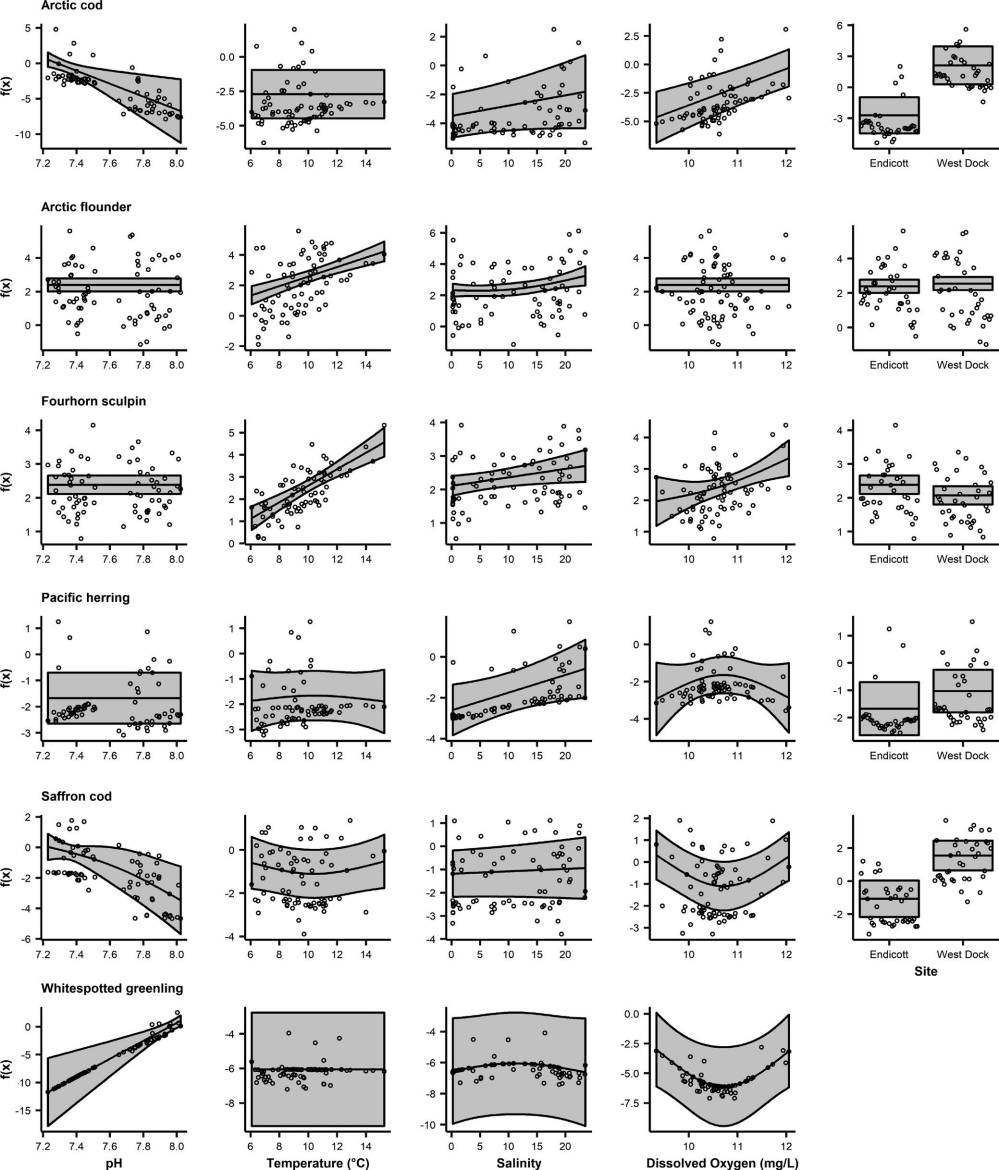
Fits of GAM covariates including 95% confidence bounds for marine species. Note that the environmental covariates were smooth terms while site is parametric

Marine fish abundance was associated with all covariates (Figure 7; Table 3). The GAM for Arctic cod (*Boreogadus saida*) explained 61% of the variance in CPUE (Table 3). Arctic cod CPUE was significantly associated linearly with pH and dissolved oxygen (Figure 7). pH was negatively associated with CPUE while dissolved oxygen was positively associated with CPUE (Figure 7). Arctic cod CPUE was higher at West Dock than Endicott (Figure 7). The variance in Arctic flounder CPUE (28%) was explained by the GAM (Table 3). Arctic flounder CPUE was significantly associated linearly with temperature and nonlinearly with salinity (Figure 7). CPUE increased with temperature and salinity (Figure 7). The GAM explained 51% of the variance in CPUE for fourhorn sculpin (*Myoxocephalus quadricornis*) (Table 3). Fourhorn sculpin CPUE was significantly associated linearly with temperature and salinity and nonlinearly with dissolved oxygen (Figure 7). Catch‐per‐unit‐effort was positively associated with all three significant covariates (Figure 7). Variance in Pacific herring CPUE (23%) was explained by the GAM (Table 3). Pacific herring CPUE increased significantly with salinity in a linear manner (Figure 7). For saffron cod (*Eleginus gracilis*), the GAM explained 42% of the variance in CPUE (Table 3). Saffron cod CPUE was significantly associated nonlinearly with pH and dissolved oxygen (Figure 7). Catch‐per‐unit‐effort decreased with pH and dissolved oxygen until ~10.8 mg/L, above which CPUE began to increase (Figure 7). Catch‐per‐unit‐effort was significantly higher at West Dock than Endicott for saffron cod (Figure 7). Seventy‐nine percent of the variance in whitespotted greenling CPUE was explained by the GAM (Table 3). Whitespotted greenling CPUE was significantly associated linearly with pH and nonlinearly with dissolved oxygen (Figure 7). Catch‐per‐unit‐effort increased with pH and decreased with dissolved oxygen until ~10.8 mg/L when the relationship became positive (Figure 7).

## 4 DISCUSSION

Spatiotemporally and taxonomically narrow characterization of the abiotic drivers of the abundance of nearshore Arctic fishes limits the ability of scientists and managers to assess the potential consequences of climate change holistically in a region that is rapidly being reshaped by environmental change. This research advances knowledge of the drivers of abundance at a fine temporal resolution for a suite of nearshore Arctic fishes of varying life histories (e.g., freshwater vs. marine). Despite its novelty, our data set is limited spatially and temporally because we only sampled during a short period of the ice‐free season at two sites, which hampers the ability to completely quantify habitat preferences. Nonetheless, we found that the abiotic environment influences the abundance of nearshore Arctic fish at a fine temporal resolution. Notably, pH was a significant predictor of abundance for six species. Further, results indicated potential affinities for different abiotic habitats displayed by Arctic fish in the nearshore. Our results suggested that fine resolution observations of the physicochemical environment should be paired with fish catch records to augment current data in order to better understand nearshore Arctic fish ecology in a changing environment.

The findings of this study indicated possible preferences for certain physicochemical conditions which could influence the abundance of species in select habitats, although it is impossible to definitively attribute these findings to habitat preference due to the limitations of our data set. Two anadromous species, Dolly Varden and pink salmon, were more abundant at low and high salinities, as indicated by the GAMs (Figure 5), which is consistent with their propensity to move between freshwater and marine environments. Arctic flounder, a stenohaline marine species which prefer high salinity (Griffiths et al., 1998), were caught in greater abundance at West Dock (Figure 3), the high salinity site, and the GAM found CPUE to increase with salinity (Figure 7). These examples highlight how the physicochemical environment can lead to differences in spatial manifestations of species occurrences and abundance in the coastal Arctic, even over short time scales. However, there are caveats which should be contemplated when considering abundance. For instance, migratory behavior and prey availability, which our study did not examine, could influence the timing of fish presence at sampling locations. Additionally, site‐specific conditions, such as currents and proximity to rivers occupied by diadromous and freshwater fishes, may influence these results. Despite these caveats, it is well established that climate envelopes and habitat preferences play a major role in influencing fish distributions and abundances in the coastal Arctic (Griffiths et al., 1998; Priest et al., 2018; Reist et al., 2006). Although we found that abundance data and GAMs reflected known associations between fish and the abiotic environment based on life history and previously described habitat preferences, these data need to be expanded to include more sites and years to fully characterize habitat preference.

Our findings indicate that Arctic fish species display disparate responses to the nearshore environment on short time scales, which may have implications for future fish community structure. Records from the BSNLTFMP sites indicated that the maximum temperatures observed during 2019 were among some of the highest observed, and are consistent with increasing average temperatures in the region since 1985. A maximum temperature of 22.1°C was observed in 2009 (Gatt et al., 2019), which is higher than the 17.2°C maximum temperature observed in 2019. Nonetheless, 2019 was the sixth warmest year on record for the BSNLTFMP (Gatt et al., 2019). These patterns of temperature maxima suggest that although 2019 was not anomalously warm, fishes in the coastal Arctic are likely experiencing extreme temperature events periodically during summer months, which could challenge their thermal tolerance, ultimately resulting in thermal stress (Somero, 2005, 2010). The 1985 and 2017 average temperatures were 6.6 and 9.5°C, respectively, for an average yearly temperature increase of 0.08°C/year (Griffiths et al., 1998; Priest et al., 2018). During our 2019 study, we recorded average temperatures of 9.4°C at Endicott and 8.7°C at West Dock, which are consistent with records of rising temperatures since the early 1980s. Concomitant changes to nearshore fish communities at the BSNLTFMP sites have been observed alongside increases in ocean temperature.

Priest et al. (2018) showed that sockeye salmon (*Oncorhynchus nerka*) and whitespotted greenling were captured by the monitoring program for the first time since its inception (in 1981) during 2017, including repeated occurrence of whitespotted greenling during 2019 (Gatt et al., 2019), which is indicative of environmental conditions supporting the persistence of emergent species after their original occurrence (Arvedlund, 2009; Fogarty et al., 2017). Annual CPUE of least cisco has decreased over time, while CPUE of saffron cod, known by previous work to be a warm tolerant species, has increased (Gatt et al., 2019; Hamman et al., 2020; Priest et al., 2018). In contrast, we found that least cisco abundance was highest at warm temperatures and saffron cod abundance was not significantly associated with temperature. These examples highlight the importance of expanding on and coupling coarse and fine scale studies to tease apart what processes are shaping fish communities in the short term and long term. Priest (2020) found that annual species richness in the nearshore Beaufort Sea, driven by temperature and salinity changes, has significantly increased by an average of one additional species per decade since 2001. These composition changes were found to benefit generalist eurythermal and euryhaline species, such as saffron cod (Priest, 2020). Signs of environmentally driven fish community restructuring can also be attributed to an increase in nonindigenous fishes in the Arctic since the 1960s (Chan et al., 2019). Indeed, these examples highlight how rapid Arctic change is altering nearshore fish assemblages and suggests that abiotic habitat preferences play a pivotal role in reshaping nearshore Arctic fish communities.

Fishes without a significant response to temperature such as Arctic cod, Arctic grayling, burbot, Pacific herring, round whitefish, saffron cod, and whitespotted greenling may also be more resilient under warming conditions. The lack of a significant response to temperature may be indicative of the acclimation of Arctic fishes to warming. For instance, Arctic cod are known to tolerate temperatures up to 13.5°C (Marsh & Mueter, 2020), yet maximum temperatures at BSNLTFMP sampling sites have been consistently warmer (Gatt et al., 2019), as was the case during this study. As climate change progresses, fishes tolerant of a wide range of physicochemical habitat conditions are more likely to persist (Ofori et al., 2017; Reist et al., 2006). Models indicate that thermal safety margins (the difference between the upper thermal tolerance limit and the maximum habitat temperature during summer) for upwards of 60% of marine and freshwater fish species could be exceeded under future climate change scenarios (Dahlke et al., 2020). In short, fishes identified in this study demonstrated disparate responses to the abiotic environment, which likely has implications for future nearshore Arctic fish assemblage composition.

To truly understand the incipient impacts a changing Arctic will have on nearshore fish communities, it is imperative that the relationships between fish and the abiotic environment are fully characterized. This study is a foundational link in understanding how the abundance of fish is driven by physicochemical habitat characteristics on a day‐to‐day basis. Expanding on these data and previously conducted coarse‐scale studies with fine resolution multivariate environmental and fish catch data collected across more sites and years is a logical next step. This would allow for interannual comparisons of fish assemblages and the potential to capture the emergence of new species in the region in relation to multivariate oceanographic data. Collecting data from a larger geographic area and across more years would provide a better characterization of habitat preference ranges by catching fish across a wider range of their preferred habitats. Further, physicochemically influenced day‐to‐day fluctuations in abundance which are truly indicative of habitat preference that may act as indicators for long‐term ecological change would become disentangled from other processes. Baseline work to characterize habitat preference ranges of nearshore Arctic fishes should also be expanded to other regions of the Arctic, as our work is limited to only a small subset of the taxa present in the coastal Arctic (Reist et al., 2006). Although it would be advantageous to extend monitoring beyond the summer field season, allowing for a longer SeaFET™ conditioning period and the capture of year‐long carbonate chemistry dynamics, ice conditions in the shallow nearshore environment currently prohibit such operations. Deploying oceanographic instrumentation in deeper regions and utilizing sampling methodologies with a demonstrated efficacy under ice, which can be deployed at a high frequency, such as environmental DNA (Khalsa et al., 2020), gill nets (Svenning et al., 2007), long lines (Cott et al., 2013), video cameras (Mueller et al., 2006), and acoustics (Mueller et al., 2006), may be a way to expand monitoring efforts.

## 5 CONCLUSION

We coupled high‐frequency in situ measurements of pH, temperature, salinity, and dissolved oxygen with daily fish catches in the nearshore Arctic to characterize the abiotic drivers of abundance occurring on a fine scale. The results of this study demonstrated that species show a varying affinity to different environmental conditions, which are consistent with life‐history and previous studies. These results indicated potential restructuring of nearshore fish communities driven by environmental change and habitat preference. We demonstrated that although certain parameters are easier to measure than others, it is worthwhile to include a wider variety of potential covariates in ecological monitoring studies as drivers of species abundance, that is, pH (a significant predictor of abundance for six species) in order to establish a comprehensive baseline understanding of the nearshore environment. Lastly, this work highlights the value of sampling fishes at a high frequency to better understand Arctic fish ecology, which has historically been informed by trawl survey, permitting a more holistic picture of potential fish community restructuring as a result of Arctic change.

## CONFLICT OF INTEREST

No conflicts of interest are declared.

## AUTHOR CONTRIBUTIONS

**Noah S. Khalsa:** Conceptualization (lead); Formal analysis (lead); Investigation (equal); Writing‐original draft (lead); Writing‐review & editing (lead). **Kyle P. Gatt:** Formal analysis (supporting); Investigation (equal); Writing‐review & editing (equal). **Trent M. Sutton:** Conceptualization (equal); Formal analysis (supporting); Writing‐review & editing (equal). **Amanda L. Kelley:** Conceptualization (equal); Formal analysis (equal); Investigation (equal); Writing‐original draft (supporting); Writing‐review & editing (equal).

## Supporting information

Supplementary Material

Supplementary Material

## Data Availability

All data used in analyses are available in a Dryad repository at the following DOI: https://doi.org/10.5061/dryad.h70rxwdjs.

## References

[ece37940-bib-0001] Arvedlund, M. (2009). First records of unusual marine fish distributions—Can they predict climate changes? Journal of the Marine Biological Association of the United Kingdom, 89, 863–866.

[ece37940-bib-0002] Beever, E. A., Hall, L. E., Varner, J., Loosen, A. E., Dunham, J. B., Gahl, M. K., Smith, F. A. et al (2017). Behavioral flexibility as a mechanism for coping with climate change. Frontiers in Ecology and the Environment, 15, 299–308.

[ece37940-bib-0004] Bonsell, C., & Dunton, K. H. (2018). Long‐term patterns of benthic irradiance and kelp production in the central Beaufort Sea reveal implications of warming for Arctic inner shelves. Progress in Oceanography, 162, 160–170. 10.1016/j.pocean.2018.02.016

[ece37940-bib-0005] Bopp, L., Resplandy, L., Orr, J. C., Doney, S. C., Dunne, J. P., Gehlen, M., Halloran, P., Heinze, C., Ilyina, T., Séférian, R., Tjiputra, J., & Vichi, M. (2013). Multiple stressors of ocean ecosystems in the 21st century: Projections with CMIP5 models. Biogeosciences, 10, 6225–6245.

[ece37940-bib-0006] Breheny, P., & Burchett, W. (2017). Visualization of regression models using visreg. The R Journal, 9, 56–71.

[ece37940-bib-0007] Cattano, C., Claudet, J., Domenici, P., & Milazzo, M. (2018). Living in a high CO_2_ world: A global meta‐analysis shows multiple trait‐mediated fish responses to ocean acidification. Ecological Monographs, 88, 320–335.

[ece37940-bib-0008] Chan, F. T., Stanislawczyk, K., Sneekes, A. C., Dvoretsky, A., Gollasch, S., Minchin, D., David, M. et al (2019). Climate change opens new frontiers for marine species in the Arctic: Current trends and future invasion risks. Global Change Biology, 25, 25–38. 30295388 10.1111/gcb.14469 PMC7379606

[ece37940-bib-0009] Cott, P. A., Johnston, T. A., & Gunn, J. M. (2013). Sexual dimorphism in an under‐ice spawning fish: The burbot (*Lota lota*). Canadian Journal of Zoology, 91, 732–740.

[ece37940-bib-0010] Craig, P. C., Griffiths, W. B., Haldorson, L., & McElderry, H. (1982). Ecological studies of Arctic cod (*Boreogadus saida*) in Beaufort Sea coastal waters, Alaska. Canadian Journal of Fisheries and Aquatic Sciences, 39, 395–406.

[ece37940-bib-0011] Dahlke, F. T., Wohlrab, S., Butzin, M., & Pörtner, H.‐P. (2020). Thermal bottlenecks in the life cycle define climate vulnerability of fish. Science, 369, 65–70. 32631888 10.1126/science.aaz3658

[ece37940-bib-0012] Dormann, C. F., Elith, J., Bacher, S., Buchmann, C., Carl, G., Carré, G., Marquéz, J. R. G., Gruber, B., Lafourcade, B., Leitão, P. J., Münkemüller, T., McClean, C., Osborne, P. E., Reineking, B., Schröder, B., Skidmore, A. K., Zurell, D., & Lautenbach, S. (2013). Collinearity: A review of methods to deal with it and a simulation study evaluating their performance. Ecography, 36, 27–46. 10.1111/j.1600-0587.2012.07348.x

[ece37940-bib-0013] Fogarty, H. E., Burrows, M. T., Pecl, G. T., Robinson, L. M., & Poloczanska, E. S. (2017). Are fish outside their usual ranges early indicators of climate‐driven range shifts? Global Change Biology, 23, 2047–2057. 28122146 10.1111/gcb.13635

[ece37940-bib-0014] Gatt, K. P., Hamman, C. R., Priest, J. T., Green, D. G., & Sutton, T. M. (2019). Beaufort Sea nearshore fish monitoring study: 2019 annual report. Report for Hilcorp Alaska, LLC by the University of Alaska Fairbanks, College of Fisheries and Ocean Sciences, Department of Fisheries.

[ece37940-bib-0015] George, C., Moulton, L., & Johnson, M. (2009). A field guide to the common fishes of the North Slope of Alaska (pp. 98). North Slope Borough, Department of Wildlife Management.

[ece37940-bib-0016] Griffiths, W. B., Fechhelm, R. G., Gallaway, B. J., Martin, L. R., & Wilson, W. J. (1998). Abundance of selected fish species in relation to temperature and salinity patterns in the Sagavanirktok Delta, Alaska, following construction of the Endicott causeway. Arctic, 51, 94–104.

[ece37940-bib-0017] Griffiths, W. B., Gallaway, B. J., Gazey, W. J., & Dillinger, R. E. Jr (1992). Growth and condition of Arctic cisco and broad whitefish as indicators of causeway‐induced effects in the Prudhoe Bay region, Alaska. Transactions of the American Fisheries Society, 121, 557–577.

[ece37940-bib-0018] Hamman, C. R., Gatt, K. P., & Sutton, T. M. (2020). Beaufort Sea nearshore fish monitoring study: 2020 annual report. Report for Hilcorp Alaska. LLC by the University of Alaska Fairbanks, College of Fisheries and Ocean Sciences, Department of Fisheries.

[ece37940-bib-0019] Harrison, X. A., Donaldson, L., Correa‐Cano, M. E., Evans, J., Fisher, D. N., Goodwin, C. E. D., Robinson, B. S. et al (2018). A brief introduction to mixed effects modelling and multi‐model inference in ecology. PeerJ, 6, e4794. 29844961 10.7717/peerj.4794 PMC5970551

[ece37940-bib-0020] Heuer, R. M., & Grosell, M. (2014). Physiological impacts of elevated carbon dioxide and ocean acidification on fish. American Journal of Physiology‐Regulatory, Integrative and Comparative Physiology, 307, R1061–R1084. 10.1152/ajpregu.00064.2014 25163920

[ece37940-bib-0021] Iken, K., Mueter, F., Grebmeier, J. M., Cooper, L. W., Danielson, S. L., & Bluhm, B. A. (2019). Developing an observational design for epibenthos and fish assemblages in the Chukchi Sea. Deep Sea Research Part II: Topical Studies in Oceanography, 162, 180–190.

[ece37940-bib-0022] Kelley, A. L., & Lunden, J. J. (2017). Meta‐analysis identifies metabolic sensitivities to ocean acidification Running title: Ocean acidification impacts metabolic function. AIMS Environmental Science, 4, 709–729.

[ece37940-bib-0023] Khalsa, N. S., Smith, J., Jochum, K., Savory, G., & López, J. A. (2020). Identifying under‐ice overwintering locations of juvenile Chinook salmon by using environmental DNA. North American Journal of Fisheries Management, 40, 762–772.

[ece37940-bib-0024] Logerwell, E., Rand, K., Danielson, S., & Sousa, L. (2018). Environmental drivers of benthic fish distribution in and around Barrow Canyon in the northeastern Chukchi Sea and western Beaufort Sea. Deep Sea Research Part II: Topical Studies in Oceanography, 152, 170–181.

[ece37940-bib-0025] Marsh, J. M., & Mueter, F. J. (2020). Influences of temperature, predators, and competitors on polar cod (*Boreogadus saida*) at the southern margin of their distribution. Polar Biology, 43, 995–1014.

[ece37940-bib-0026] Mecklenberg, C. W., Mecklenberg, T. A., & Thorsteinson, L. K. (2002). Fishes of Alaska (Vol. 1, pp. 116). American Fisheries Society.

[ece37940-bib-0027] Miller, C. A., Bonsell, C., McTigue, N. D., & Kelley, A. L. (2021). The seasonal phases of an Arctic lagoon reveal the discontinuities of pH variability and CO_2_ flux at the air–sea interface. Biogeosciences, 18, 1203–1221.

[ece37940-bib-0028] Miller, C. A., & Kelley, A. L. (2021). Seasonality and biological forcing modify the diel frequency of nearshore pH extremes in a subarctic Alaskan estuary. Limnology and Oceanography, 66, 1475–1491.

[ece37940-bib-0029] Miller, C. A., Pocock, K., Evans, W., & Kelley, A. L. (2018). An evaluation of the performance of Sea‐Bird Scientific's SeaFET™ autonomous pH sensor: Considerations for the broader oceanographic community. Ocean Science, 14, 751–768. 10.5194/os-14-751-2018

[ece37940-bib-0030] Mueller, R. P., Brown, R. S., Hop, H., & Moulton, L. (2006). Video and acoustic camera techniques for studying fish under ice: A review and comparison. Reviews in Fish Biology and Fisheries, 16, 213–226.

[ece37940-bib-0031] Mueter, F. J., & Litzow, M. A. (2008). Sea ice retreat alters the biogeography of the Bering Sea continental shelf. Ecological Applications, 18, 309–320. 18488598 10.1890/07-0564.1

[ece37940-bib-0032] Munday, P., Pratchett, M., Dixson, D., Donelson, J., Endo, G., Reynolds, A., & Knuckey, R. (2013). Elevated CO_2_ affects the behavior of an ecologically and economically important coral reef fish. Marine Biology, 160, 2137–2144.

[ece37940-bib-0033] Ofori, B. Y., Stow, A. J., Baumgartner, J. B., & Beaumont, L. J. (2017). Influence of adaptive capacity on the outcome of climate change vulnerability assessment. Scientific Reports, 7, 12979. 29021590 10.1038/s41598-017-13245-y PMC5636830

[ece37940-bib-0034] Overland, J. E., Wang, M., Walsh, J. E., & Stroeve, J. C. (2014). Future Arctic climate changes: Adaptation and mitigation time scales. Earth's Future, 2, 68–74.

[ece37940-bib-0035] Priest, J. T. (2020). Long‐term shifts in community structure, growth, and relative abundance of nearshore Arctic fishes: A response to changing environmental conditions. Unpublished master's thesis, University of Alaska Fairbanks.

[ece37940-bib-0036] Priest, J. T., Green, D. G., Fletcher, B. M., & Sutton, T. M. (2018). Beaufort Sea nearshore fish monitoring study: 2017 annual report. Report for Hilcorp Alaska, LLC by the University of Alaska Fairbanks, College of Fisheries and Ocean Sciences, Department of Fisheries.

[ece37940-bib-0037] Qi, D., Chen, L., Chen, B., Gao, Z., Zhong, W., Feely, R. A., Anderson, L. G. et al (2017). Increase in acidifying water in the western Arctic Ocean. Nature Climate Change, 7, 195–199.

[ece37940-bib-0038] R Core Team (2018). R: A language and environment for statistical computing. R Foundation for Statistical Computing.

[ece37940-bib-0039] Reist, J. D., Wrona, F. J., Prowse, T. D., Power, M., Dempson, J. B., Beamish, R. J., King, J. R. et al (2006). General effects of climate change on Arctic fishes and fish populations. AMBIO: A Journal of the Human Environment, 35, 370–380. 10.1579/0044-7447(2006)35[370:geocco]2.0.co;2 17256641

[ece37940-bib-0040] Shu, Q., Qiao, F., Song, Z., Zhao, J., & Li, X. (2018). Projected freshening of the Arctic Ocean in the 21st century. Journal of Geophysical Research: Oceans, 123, 9232–9244.

[ece37940-bib-0041] Somero, G. N. (2005). Linking biogeography to physiology: Evolutionary and acclimatory adjustments of thermal limits. Frontiers in Zoology, 2. 10.1186/1742-9994-2-1 PMC546413 15679952

[ece37940-bib-0042] Somero, G. N. (2010). The physiology of climate change: How potentials for acclimatization and genetic adaptation will determine ‘winners’ and ‘losers’. The Journal of Experimental Biology, 213, 912–920. 20190116 10.1242/jeb.037473

[ece37940-bib-0043] Svenning, M.‐A., Klemetsen, A., & Olsen, T. (2007). Habitat and food choice of Arctic charr in Linnévatn on Spitsbergen, Svalbard: The first year‐round investigation in a High Arctic lake. Ecology of Freshwater Fish, 16, 70–77.

[ece37940-bib-0044] Thorsteinson, L. K., & Love, M. S. (Eds.) 2016. Alaska Arctic marine fish ecology catalog: U.S. Geological Survey Scientific Investigations Report 2016‐5038 (OCS Study, BOEM 2016‐048). (pp. 768).Reston, VA: U.S. Geological Survey.

[ece37940-bib-0045] Wassmann, P., Duarte, C. M., Agustí, S., & Sejr, M. K. (2011). Footprints of climate change in the Arctic marine ecosystem. Global Change Biology, 17, 1235–1249.

[ece37940-bib-0046] Wickham, H. (2016). ggplot2: Elegant graphics for data analysis. Springer‐Verlag.

[ece37940-bib-0047] Wood, S. A. (2017). Generalized additive models: An introduction with R (2nd ed.). Chapman & Hall/CRC.

[ece37940-bib-0048] Zeller, D., Booth, S., Pakhomov, E., Swartz, W., & Pauly, D. (2011). Arctic fisheries catches in Russia, USA, and Canada: Baselines for neglected ecosystems. Polar Biology, 34, 955–973. 10.1007/s00300-010-0952-3

