# Supplementary material for: Characterization of the abiotic drivers of abundance of nearshore Arctic fishes
Source: Ecol Evol. 2021 Jul 22;11(16):11491–506. doi: 10.1002/ece3.7940 (PMC8366885; doi:10.1002/ece3.7940)
Supplement: Supplementary file 1 — Supplementary Material [file ECE3-11-11491-s001.docx]

*Sensor Calibration.—*

Discrete water samples for SeaFET™ calibration and reference samples were collected in duplicate, proximal to each sensor array just below the water surface. Water sampling occurred within 30 seconds of the instrument sampling period. For Endicott, discrete water samples were collected on 4 July 2019, 16:00, 5 July 2019, 20:00, 6 July 2019, 16:00, and 21 August 2019, 22:00 UTC. All four samples were used for multipoint calibration of pH values, and all discrete water samples were used as references to examine sensor uncertainty. Discrete water samples for West Dock were collected following the same methods on 4 July 2019, 19:00, 5 July 2019, 17:00, 6 July 2019, 18:00, and 21 July 2019, 17:00 UTC. The 4 July 2019 sample was used to calibrate sensor pH values, and the following discrete samples were used to quantify pH uncertainty for the West Dock sensor. Samples were stored in 500-mL borosilicate bottles, pre-spiked with 200-µL saturated mercuric chloride at ~ 4.0℃ until laboratory analysis. We used a Shimadzu 1800 spectrophotometer at 25.0℃ to measure pH for each sample (spectrophotometric method, SOP 6b, Dickson et al. 2007; using *meta*-cresol purple from Acros, batch # 30AXM-QN). A dye impurity correction factor (Douglas and Byrne, 2017) was then applied to the final calculation of pH. Total alkalinity (A_T_) was measured via open-cell titration (A_T_, SOP 3b, Dickson et al. 2007) with a Metrohm 848 Titrino plus. At the time of sample collection, a handheld digital thermometer (Omega, HH81A) was used to record temperature. We used CO2Calc with constants from Lueker et al. (2000), Uppström (1974), and Dickson et al. (1990) and with input parameters of spectrophotometric pH (25.0℃) and A_T_ to calculate pH_T_. Voltage measurements recorded by the SeaFET™ were converted to pH_T_ as described in Martz et al. (2010) using the multi-point calibration method for Endicott and the single-point calibration method for West Dock (Bresnahan et al., 2014; Miller et al., 2018). Using the lab derived *in situ* pH from the bottle samples, calibration coefficients were calculated and applied to each data set to calculate pH. Voltage measurements from the internal SeaFET™ electrode were used for Endicott and from the external SeaFET™ electrode for West Dock. Electrode choice was directed based on examination of sensor variability, stability, and accuracy over the study period. All reported pH time-series values are on the total hydrogen ion scale.

HOBO-derived salinity data and miniDOT-derived oxygen data for each site was also calibrated. Salinity data was post-processed within the HOBOware® Pro software using the 4 July 2019 and 21 August 2019 laboratory measured discrete bottle samples as endmembers for each site’s salinity dataset. A YSI 3100 conductivity meter was used to measure the salinity of calibration bottle samples used as end members for the salinity dataset. Oxygen data were calibrated within the miniDOT software using the mean discrete bottle sample salinities for each site and mean atmospheric pressure for our study period sourced from the NOAA National Data Buoy Center Station PRDA2 - 9497645 - Prudhoe Bay, AK.

*pH Uncertainty Estimates.—*

Calibration and reference bottle samples were assessed for overall accuracy of the pH time-series using propagation uncertainty according to standard operating procedures and best practices in chemical oceanography (Dickson et al., 2007; Orr et al., 2018; Miller and Kelley, 2021). Briefly, we propagated the total uncertainty associated with the pH data by combining the calculated uncertainty of our analytical measurements with the discrepancy between our calibration reference samples and measured pH. Analytical measurement uncertainty was given as:

1. $Q=\sqrt{{SD}_{m-cresol}^{2}+{SD}_{Bottle replicates}^{2}+{SD}_{Titrator}^{2}+{SD}_{CO2SYS constants}^{2}}$,

where $Q$ was the propagated uncertainty and $SD$ was the standard deviation of spectrophotometric measurement error from *m*-cresol validated via triplicate analytical precision using Certified Reference Material (CRM: Batch 172, A.G., Dickson, Scripps Institute of Oceanography), the discrete reference bottle sample replicates, A_T_ titration duplicates from bottle samples compared against the CRM A_T_, and CO2Sys constants. The calculated analytical uncertainty was combined with the difference between reference bottle pH and sensor measured pH to propagate the uncertainty for both pH time series.

Table 1. Propagated uncertainty for derived pH including the total pH uncertainty (in pH units) at Endicott and West Dock.

| Uncertainty type | Endicott | West Dock |
| --- | --- | --- |
| Bottle sample discrepancy | 0.155 | 0.252 |
| Propagated error | 0.012 | 0.020 |
| Total uncertainty | 0.167 | 0.272 |

**References**

Bresnahan, P. J., Martz, T. R., Takeshita, Y., Johnson, K. S., LaShomb, M. 2014. Best practices for autonomous measurement of seawater pH with the Honeywell Durafet. *Methods in Oceanography*, 9: 44-60.

Dickson, A. G., Sabine, C. L., Christian, J. R. 2007. Guide to best practices for ocean CO_2_ measurements. *PICES Special Publication*, 3: 191.

Dickson, A. G., Wesolowski, D. J., Palmer, D. A., Mesmer, R. E. 1990. Dissociation constant of bisulfate ion in aqueous sodium chloride solutions to 250°C. *The Journal of Physical Chemistry*, 94: 7978-7985.

Douglas, N. K., Byrne, R. H. 2017. Spectrophotometric pH measurements from river to sea: calibration of mCP for 0≤S≤40 and 278.15≤T≤308.15K. *Marine Chemistry*, 197: 64-69.

Lueker, T., Dickson, A., Keeling, C. 2000. Ocean pCO_2_ calculated from dissolved inorganic carbon, alkalinity, and equations for K1 and K2: validation based on laboratory measurements of CO_2_ in gas and seawater at equilibrium. *Marine Chemistry*, 70: 105-119.

Martz, T. R., Connery, J. G., Johnson, K. S. 2010. Testing the Honeywell Durafet® for seawater pH applications. *Limnology and Oceanography: Methods*, 8: 172-184.

Miller, C. A., Kelley, A. L. 2021. Seasonality and biological forcing modify the diel frequency of nearshore pH extremes in a subarctic Alaskan estuary. *Limnology and Oceanography*, 66: 1475-1491.

Orr, J. C., Epitalon, J.-M., Dickson, A. G., Gattuso, J.-P. 2018. Routine uncertainty propagation for the marine carbon dioxide system. *Marine Chemistry*, 207: 84-107.

Uppström, L. R. 1974. The boron/chlorinity ratio of deep-sea water from the Pacific Ocean. *Deep Sea Research and Oceanography Abstracts*, 21: 161-162.
