# Supplementary material for: Characterization of the abiotic drivers of abundance of nearshore Arctic fishes
Source: Ecol Evol. 2021 Jul 22;11(16):11491–506. doi: 10.1002/ece3.7940 (PMC8366885; doi:10.1002/ece3.7940)
Supplement: Supplementary file 2 — Supplementary Material [file ECE3-11-11491-s002.docx]

Table 1. Summary statistics for length (nearest 1 mm) composition data of selected species. All lengths are fork length, except for Arctic cod and saffron cod, which are total length. Values are reported to the nearest whole number.

|  | Endicott | | | | West Dock | | | |
| --- | --- | --- | --- | --- | --- | --- | --- | --- |
| Species | Median | Max | Min | Mean ± S.D. | Median | Max | Min | Mean ± S.D. |
| Arctic cisco | 144 | 399 | 65 | 177 ± 74 | 167 | 402 | 70 | 198 ± 71 |
| Arctic cod | 102 | 182 | 59 | 103 ± 24 | 97 | 177 | 50 | 100 ± 21 |
| Broad whitefish | 154 | 479 | 50 | 171 ± 78 | 240 | 499 | 63 | 257 ± 84 |
| Dolly Varden | 155 | 476 | 75 | 196 ± 102 | 156 | 540 | 76 | 228 ± 130 |
| Humpback whitefish | 341 | 434 | 164 | 335 ± 50 | 328 | 425 | 130 | 321 ± 59 |
| Least cisco | 262 | 376 | 75 | 251 ± 64 | 249 | 417 | 95 | 242 ± 56 |
| Pink salmon | 424 | 475 | 415 | 438 ± 32 | 425 | 495 | 392 | 434 ± 29 |
| Saffron cod | 183 | 312 | 86 | 182 ± 46 | 201 | 461 | 40 | 180 ± 95 |
